# Effects of Airway Generation Depth on Particle Deposition Behaviour in the Main Respiratory Airways—A Computational Modelling Study

**DOI:** 10.1007/s11095-026-04084-6

**Published:** 2026-04-08

**Authors:** Xinyu Cai, Damien Chong, Hui Xin Ong, Kiao Inthavong, Ann Lee, Jingliang Dong, Agisilaos Kourmatzis, Shaokoon Cheng

**Affiliations:** 1 School of Engineering, Faculty of Science and Engineering, Macquarie University, Sydney, Australia; 2 Chemical Biological Radiological and Nuclear Defence, Sensors and Effectors Division, Defence Science and Technology Group, 506 Lorimer St., Fishermans Bend, Melbourne, VIC 3207 Australia; 3 Faculty of Medicine, Healthy and Human Sciences, Macquarie University, Sydney, NSW 2109 Australia; 4 Respiratory Technology, The Woolcock Institute of Medical Research, Sydney, NSW 2113 Australia; 5 School of Aerospace, Mechanical and Manufacturing Engineering, RMIT University, Plenty Road, Bundoora, Melbourne, VIC PO Box 71, 3083 Australia; 6 Institute for Sustainable Industries & Liveable Cities, Victoria University, Melbourne, VIC PO Box 14428, 8001 Australia; 7 First Year College, Victoria University, Footscray Park Campus, Footscray, VIC 3011 Australia; 8 School of Aerospace, Mechanical and Mechatronic Engineering, The University of Sydney, Sydney, Australia

**Keywords:** computational fluid dynamics, human airway, particle deposition, realistic lower airway

## Abstract

**Purpose:**

Effective inhaled drug delivery depends on formulation, device, and a clear understanding of aerosol transport in the respiratory tract. This study explores how lower airway anatomical complexity affects airflow and particle deposition in the main respiratory airways.

**Methods:**

Seven 3D bronchial models with varying airway generational depths were developed. Numerical simulations were conducted using the discrete phase model (DPM) to simulate aerosol transport and deposition under transient inspiratory flows at inhalation peak flow rates of 60 and 120 L/min.

**Results:**

Our results showed that simplified models with three bronchial generations could underestimate the deposition efficiency in the main respiratory airways by up to 65.6% compared to the seven-generation lower airway model. Additionally, disparities in deposition outcomes and flow characteristics diminished with increasing lower airway complexity, with minimal changes in flow dynamics and deposition observed beyond the model with six bronchial generation.

**Conclusions:**

This study suggests that airway models incorporating at least up to the 6th generation of bronchial branching may be required to sufficiently capture the implications for inhalation therapy design and the development of reliable in silico testing frameworks. Notably, insufficient lower airway detail not only compromises lung deposition estimates but also affects airflow dynamics throughout the entire respiratory tract, including the upper airway. This study highlights the importance of retaining detailed geometry in the design of inhalation therapies and the development of in silico testing frameworks.

**Supplementary Information:**

The online version contains supplementary material available at https://doi.org/10.1007/s11095-026-04084-6.

## Introduction

Understanding particle deposition behaviour in the human respiratory airways is an important area of study with far-reaching implications. For example, knowledge in this area can be useful for the pharmaceutical industry, which would benefit from having insights on how a given formulation is deposited in the lower airways for effective drug delivery. In public health, particularly in the context of environmental pollution, an accurate understanding of the deposition of inhaled particles is meaningful for assessing how they may exacerbate health issues in vulnerable populations, such as those prone to respiratory infections. The economic burden associated with respiratory diseases is staggering, with an estimated annual cost of $380 billion among European countries alone, largely attributed to healthcare expenses and lost productivity [1].

Particle deposition in the respiratory airways is influenced by multiple interacting factors such as flow conditions, particle properties [2], and the geometry of the airway. While a higher flow rate imparts greater kinetic energy to the particles, potentially aiding their deeper penetration into the lungs, the tortuosity of the upper airway increases the likelihood of these particles, especially the larger ones, to impact against the airway walls, thereby reducing their propensity to reach the lower airways [3–5]. Smaller particles, on the other hand, are less affected by inertia and are more likely to reach the deeper lung regions through sedimentation and diffusion, but they come with the caveat of being more likely to be exhaled before depositing, particularly during shallow breathing.

The human respiratory tract (HRT) comprises the conducting zone and the respiratory zone. The conducting zone includes the nasal and oral cavities down to the terminal bronchioles, while the respiratory zone consists of the respiratory bronchioles and alveolar ducts. In this study, the term “lower airway” refers specifically to the tracheobronchial conducting region extending from the trachea to the seventh bronchial generation included in the computational models. The adult human lung has about 23 generations of bifurcation, with each generation exhibiting distinct structural and functional characteristics [6]. Accurate modelling of the respiratory airway is indubitably useful for capturing airflow and particle transport behaviours essential for meaningful clinical and pharmaceutical analysis. However, fairly large inter-subject variations in airway geometries, particularly in the conducting zone, have been shown to be affected by factors such as age group and anatomical features [7–9]. This highlights the importance of using airway models that strike a balance between anatomical detail and generalisability, so that simulations can be accurate and broadly applicable. Several studies have investigated the influence of airway geometric complexity on inhalation dosimetry using both experimental and numerical approaches. For example, Su *et al*. [10] experimentally examined ultrafine particle deposition in 3D-printed lung models retaining different generations of airway bifurcations, while Xi *et al*. [11] numerically analysed airflow and particle deposition in tracheobronchial geometries using statistical shape modelling. These studies demonstrate that airway geometric structure can significantly influence aerosol transport and deposition behaviour. However, the extent to which truncating the lower airway at different bronchial generations affects airflow dynamics and particle deposition in connected upper airway-lung models remains insufficiently understood.

In the existing literature, studies on particle deposition efficiency in the lower airways have used models with varying levels of complexity [12–16]. In some of these works, the lower airways are simplified, often limiting their representation to only a few generations. In the Weibel model, for example, the human lung is creatively simplified into a symmetric branching tree structure, providing a foundational framework for understanding airflow and particle transport in airway bifurcations [14]. While detailed lower airway models have also been developed [15, 17–19], whether lung deposition outcomes differ significantly between models with varying lower airway geometric complexities, particularly in terms of the number of bronchial generations represented, remains unclear.

In this study, we hypothesise that the complexity of lower airway geometries may influence upstream flow behaviour, thereby significantly affecting the overall deposition pattern along the respiratory tract. Understanding the minimal number of bronchial generations required can be useful for developing future computational models that are both efficient and sufficiently accurate in capturing particle deposition across different airway regions. This understanding is also beneficial for informing the design and manufacturing of physiologically representative lung replicas that balance structural complexity with an accepted level of practicality to facilitate fabrication and cleaning.

## Method

### Model Reconstruction

**Fig. 1:**
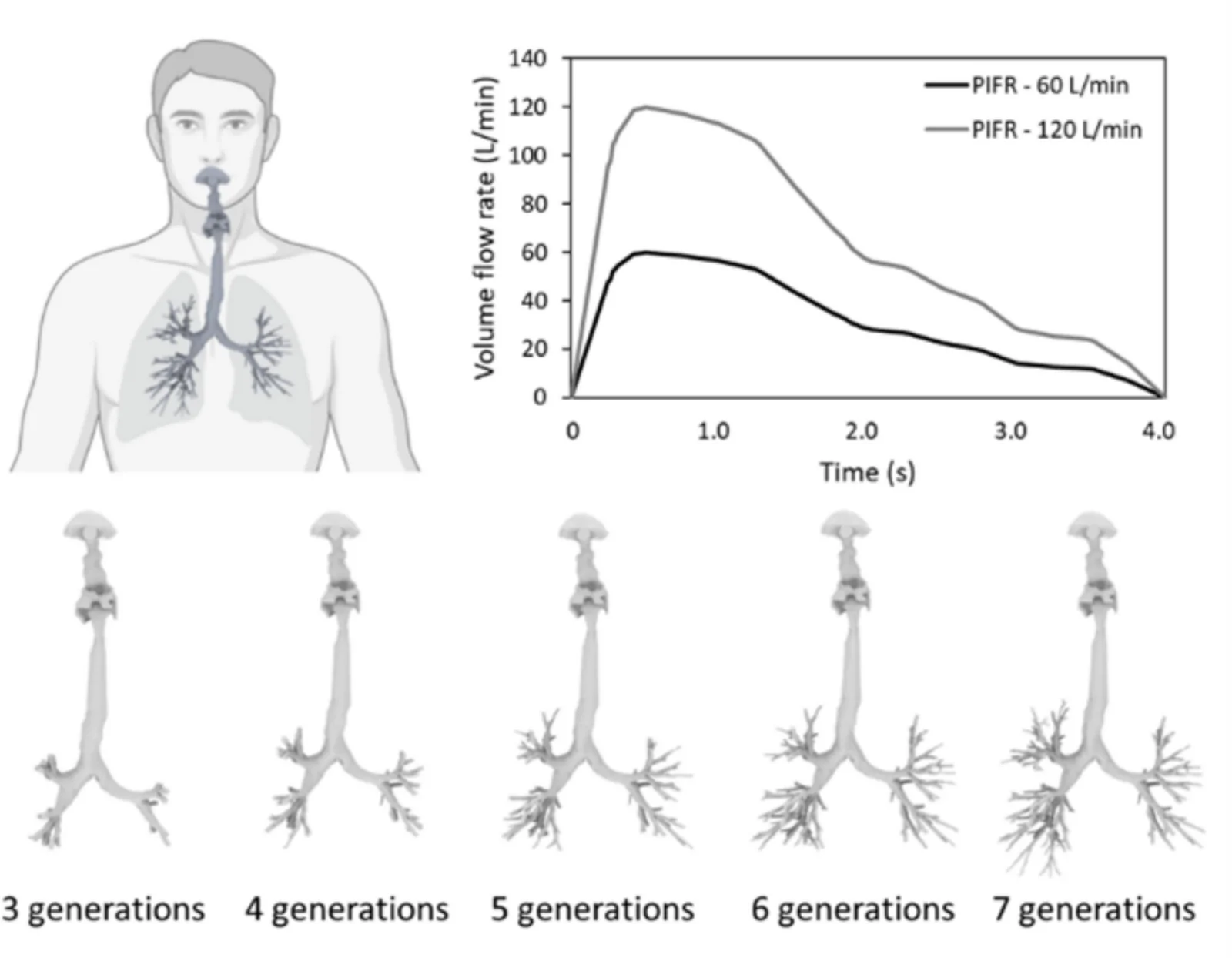
Realistic airway models with different generations and inhalation flow profiles used in this study.

A geometrically realistic upper airway model, including the trachea, was reconstructed from MRI images using 3D Slicer (www.slicer.org). The lower airway, extending to the seventh airway generation, was sourced from previous study conducted by Shang *et al*. [19], in which a subject-specific airway was reconstructed from CT images of a health Asian adult, including nasal airway passage and conducting bronchial airways up to the 15th generation. In the present study, the authors leveraged Shang’s model and redesigned it to create an airway with oral openings, retaining the lower-airway portion required for the generation-truncation analysis described herein. The connection between the upper and lower airway models was established using Rhinoceros (Rhino 7, Robert McNeel & Associates, Seattle, Washington). A cylindrical inhaler extension, with cross section modelling the outlet of the Aerolizer powder inhaler, was attached to the inlet of the upper airway model. Four other variants of the airway model, comprising of lower airways ranging from three to seven generations, were then developed from the base model to systematically evaluate the impact of airway generation on particle transport and deposition (see Fig. 1). The total outlet surface area of each airway model was also calculated to quantify the geometric differences introduced by including additional bronchial generations. The outlet surface areas for the 3rd-generation to 7th-generation lower airway models were 506.13 mm^2^, 575.61 mm^2^, 644.72 mm^2^, 650.11 mm^2^, and 667.13 mm^2^, respectively. As expected, the total outlet area increases as more distal airway branches are included in the computational domain.

**Fig. 2:**
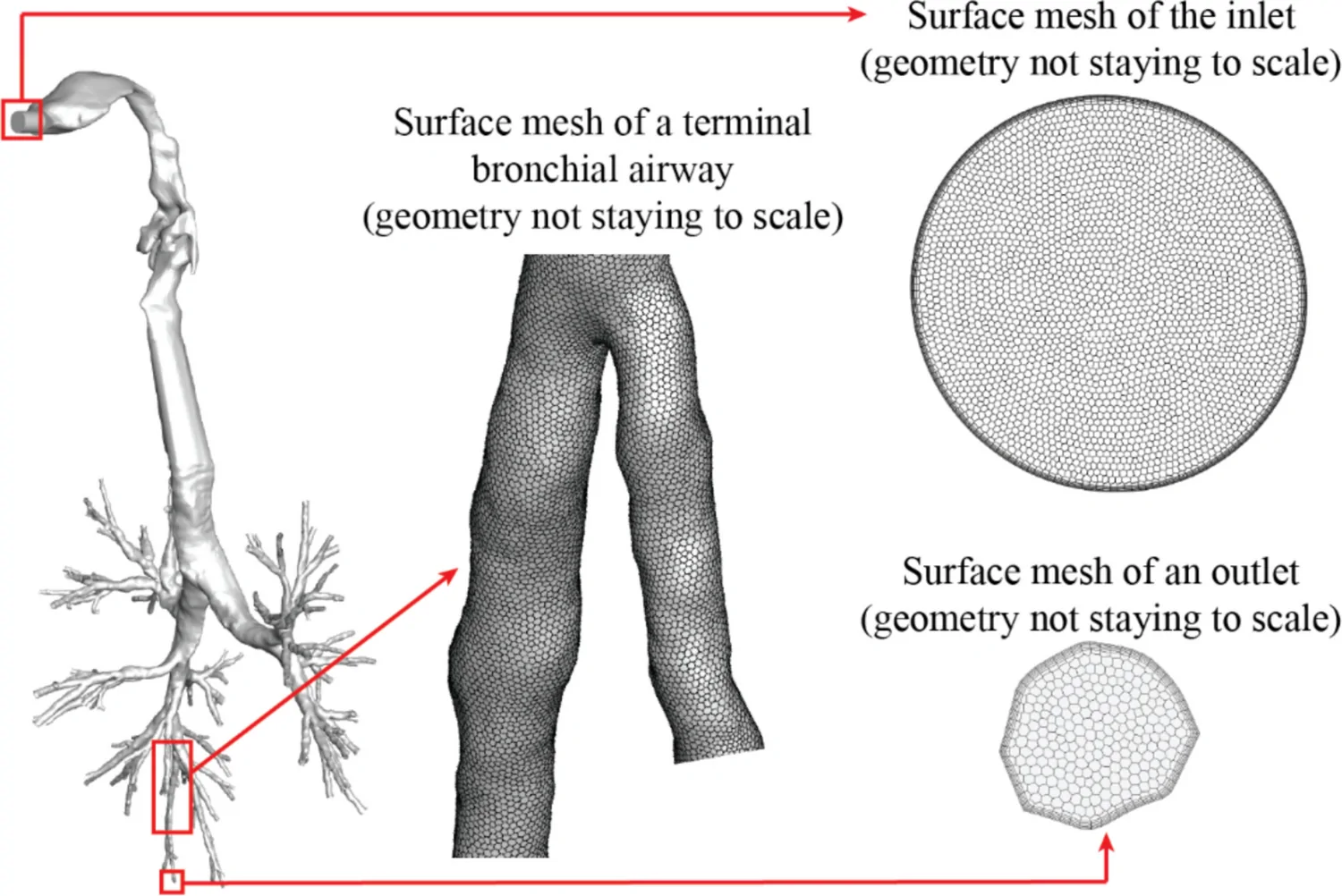
Mesh for the models (12 million mesh size).

The models were discretised into polyhedral meshes in Fluent (Ansys 2019 R3, Canonsburg, Pennsylvania) (see surface mesh; Fig. 2). Polyhedral meshes have been used in many other studies in this field due to their high mesh quality that enables computational efficiency, without compromising on accuracy [20].

As shown in Fig. 1, two transient flow profiles derived from Chrystyn [21] with peak inspiratory volume flow rate (PIFR) at 60 L/min and 120 L/min were considered and two particle sizes (3 µm and 7 µm) were included in the study design. These sizes were chosen to represent a range of aerodynamic diameters relevant to deep lung deposition (3 µm) and upper airway retention (7 µm), providing insights into size-dependent pulmonary drug delivery dynamics. Additionally, steady flow rates of 60 L/min and 120 L/min were also simulated to compare their effects with the transient flow profiles, given the body of work in the literature that has used steady-state flow conditions. These variables resulted in the development and simulation of a total of 15 computational models.

The Reynolds number in the human airway ranges from approximately 7000–15,000 in the upper regions (e.g., oral cavity, epiglottis, and inhaler mouthpiece) to below 1300 in the distal bronchioles near the seventh generation. The k-ω SST model is selected for the simulation, given its widespread use, in similar field of research [15, 22–25]. Discrete phase modelling (DPM) was performed, and properties of mannitol (density: 1514 kg/m^3^) were modelled. The boundary condition at the inlet and outlet was prescribed as escape, while the walls of the inhaler and the entire airway were prescribed as trapped condition. The turbulent intensity at the inlet was defined as 5%, which has been commonly used in existing work on RANS simulations to represent fluid motion irregularities [15, 19, 23, 26–28] in respiratory models.

**Fig. 3:**
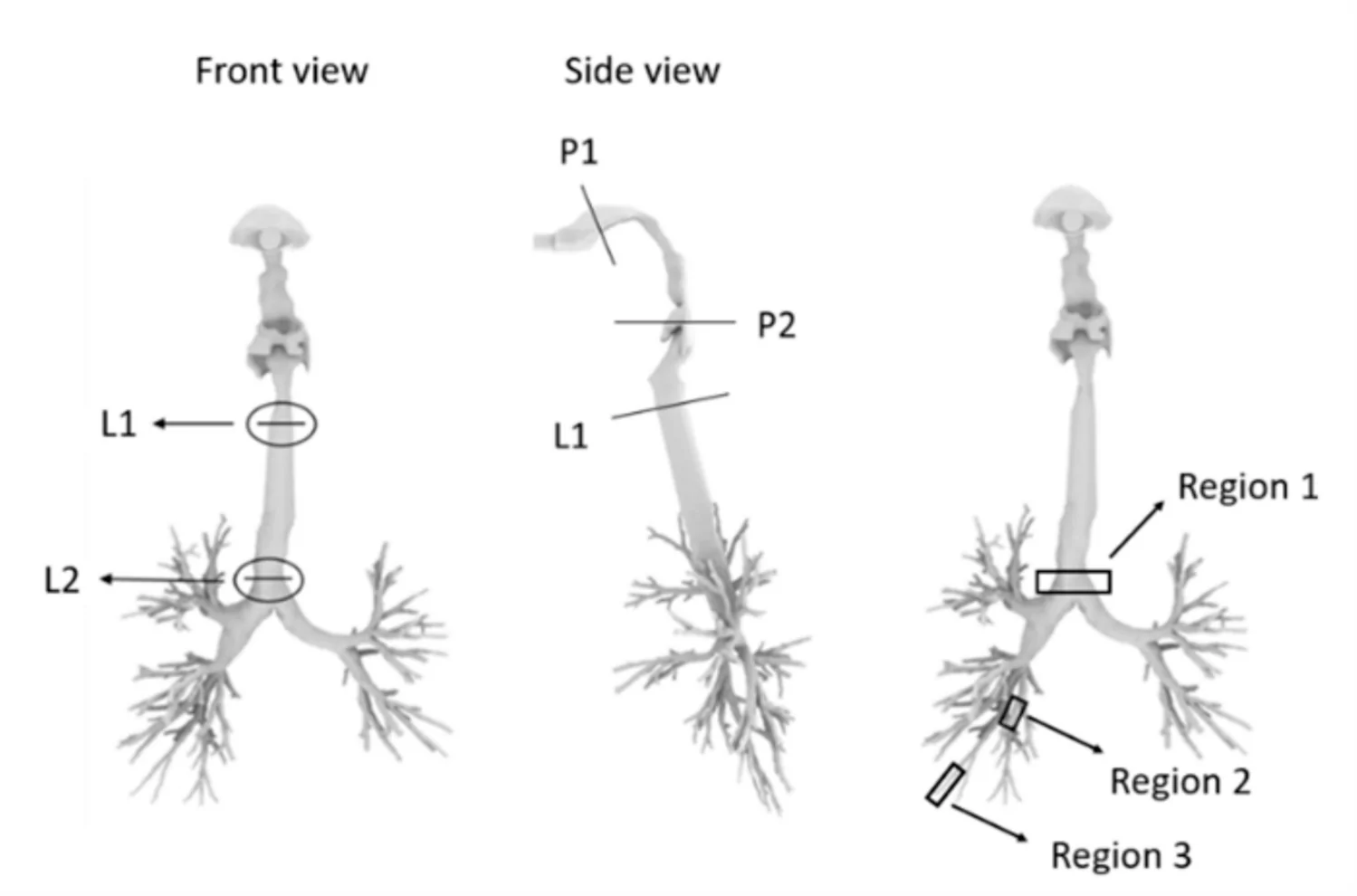
Region of interest defined for particle deposition, velocity profiles and velocity contour analysis. The 7th-generation airway model is shown as a representative example.

The following regions of interest (see Fig. 3) were defined to analyse airflow dynamics and particle deposition efficiency. Two cross-sectional planes in the pharynx were selected: P1, located in the oral cavity, and P2, at the level of the epiglottis. Additionally, two axial planes along the trachea were chosen: L1, at the tracheal inlet, to analyse velocity contours, and L2, positioned just before the airway bifurcation, to assess the axial velocity profile. For particle deposition analysis, in addition to evaluating whole-lung deposition (including particles deposited in the trachea, tracheobronchial tree, and escaped particles), three specific regions within the bronchial tree were selected: Region 1, the tracheobronchial junction (carina); Region 2, a third-generation bronchial segment; and Region 3, a terminal airway in the right lower lobe.

**Fig. 4:**
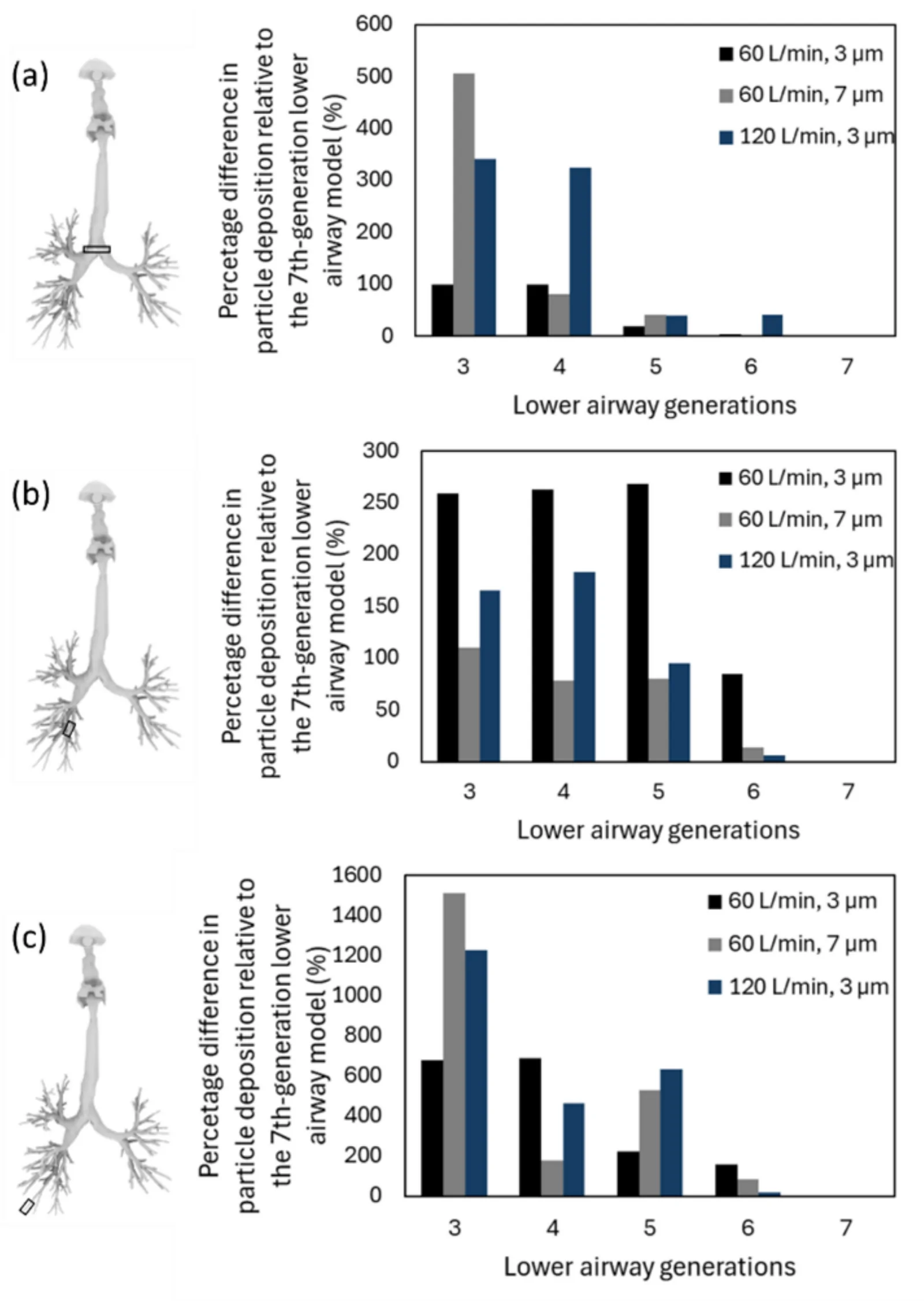
The potential effects of the number of lower airway generation on regional particle deposition under three inhalation conditions (PIFR 60 L/min—3 µm, PIFR 60 L/min—7 µm, and PIFR 120 L/min—3 µm). Each bar chart plots of relative particle deposition difference compared to the seventh-generation lower airway model.

**Fig. 5:**
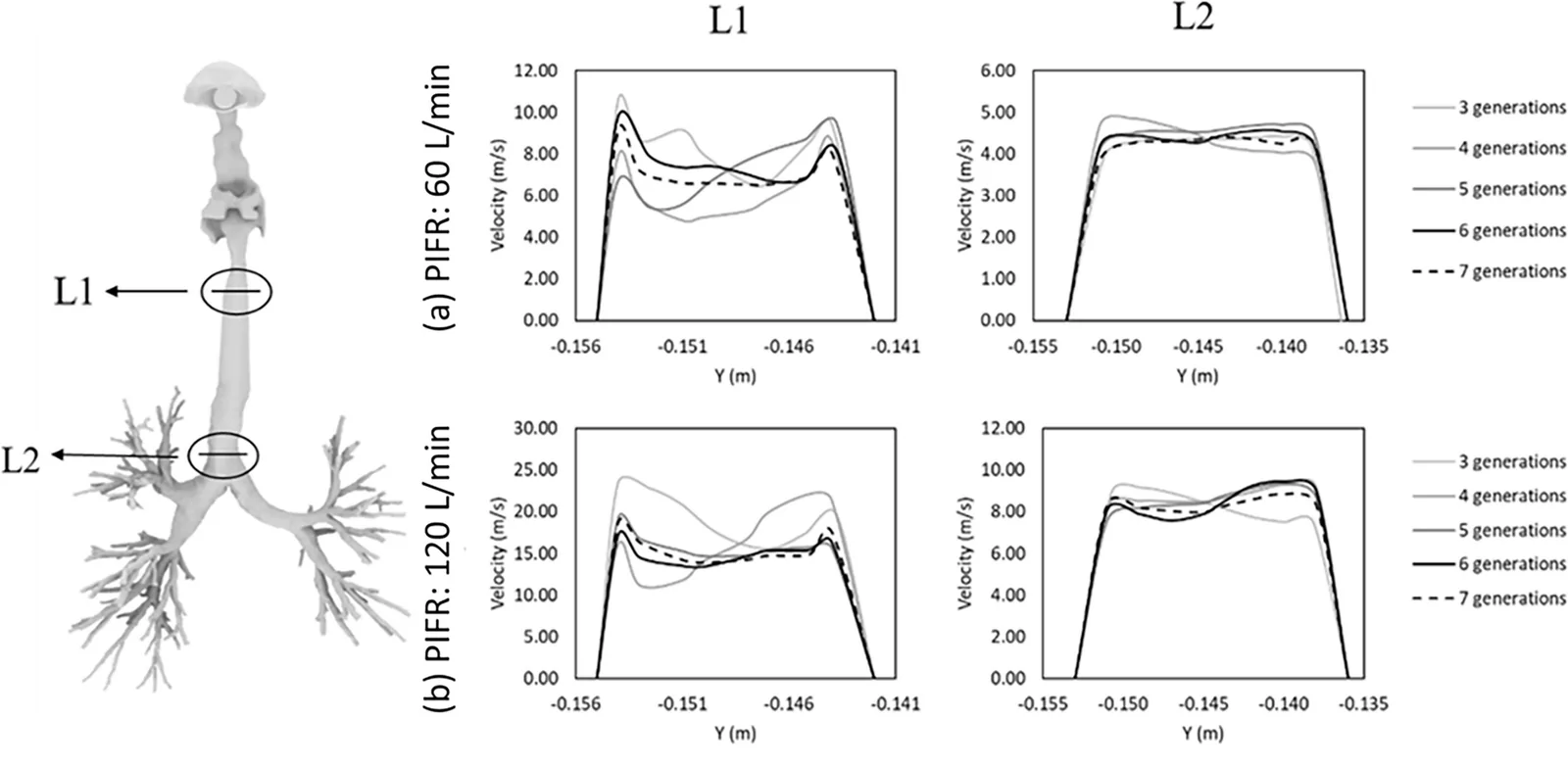
Velocity profiles along two lines across the airway cross-sections L1 (at the tracheal inlet) and L2 (positioned just before the airway bifurcation). Panel (**a**) represents cases simulated under a transient flow condition (PIFR at 60 L/min), and panel (**b**) shows results under a higher flow rate condition (PIFR at 120 L/min).

**Fig. 6:**
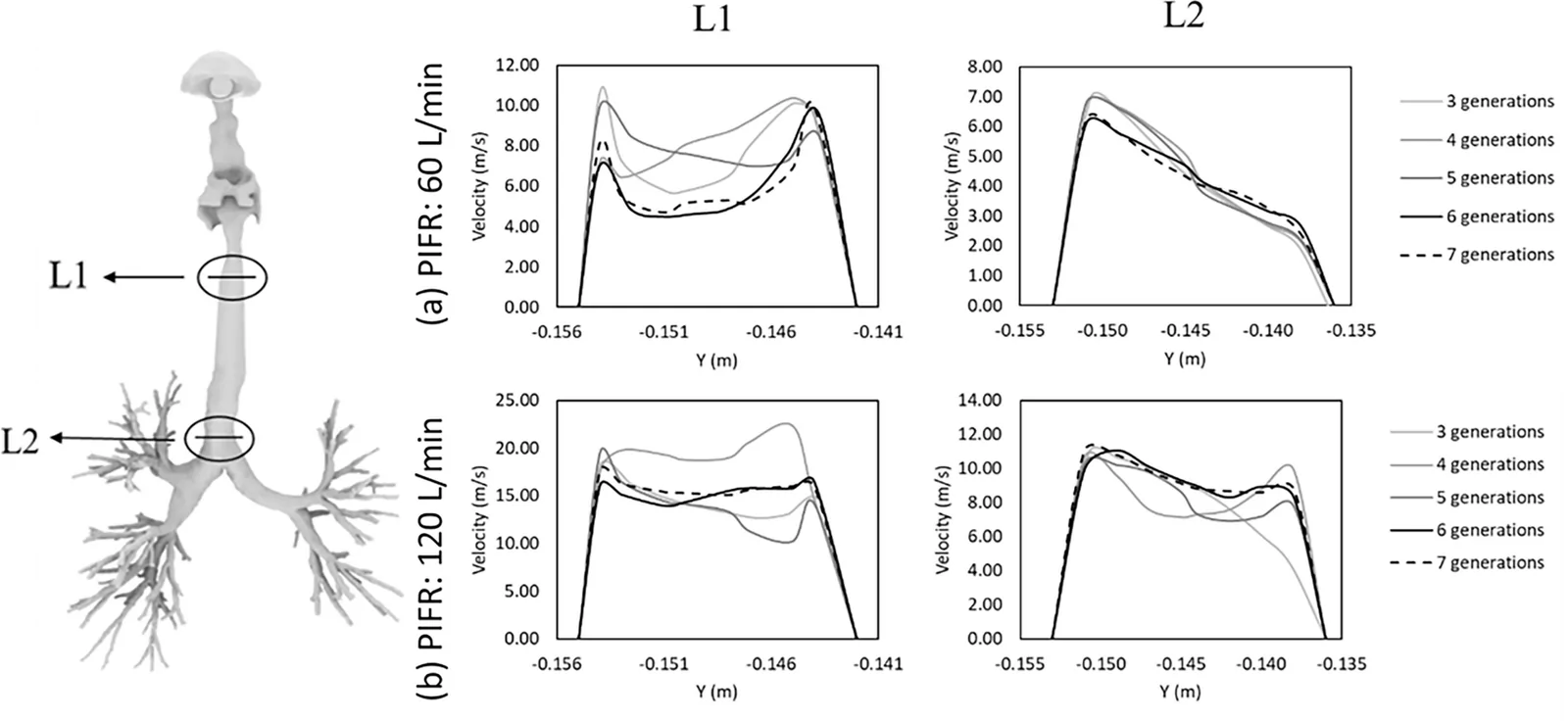
Velocity profiles along two lines across the airway cross-sections, and along the coronal plane. Panel (**a**) represents cases simulated under a steady state flow condition (60 L/min), and panel (**b**) shows results under a higher flow rate (120 L/min).

**Fig. 7:**
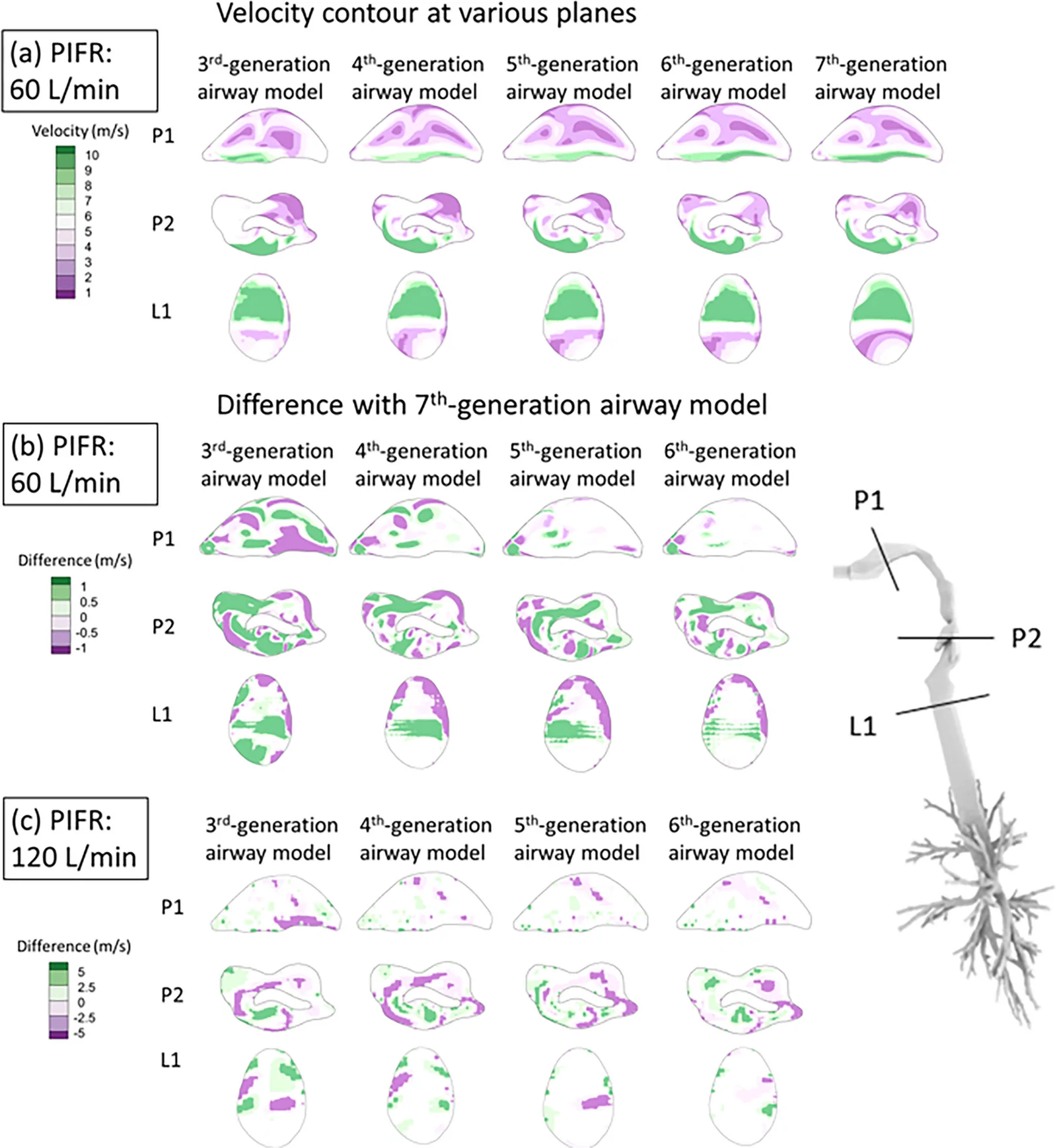
(**a**) Velocity contours for the five models with different number of lower airway generations. (**b**) Computed velocity contour difference for models simulated at PIFR of 60L/min. (**c**) Computed velocity contour difference for models simulated at PIFR of 120L/min.

To ensure that the results of this study were not influenced by boundary condition setups, two distinct outlet boundary conditions, each used in existing published work, were applied to the seventh-generation lower airway model. In the first approach (boundary condition 1), a pressure condition of *P* = 0 was prescribed at the airway outlets, consistent with previous studies [29–31], while a velocity inlet condition based on the inhalation volumetric flow rate was imposed at the model’s inlet. This pressure-outlet boundary condition was used for all simulations presented in Tables 1 - 2 and Figs. 4, 5, 6 and 7. In the second approach (boundary condition 2), lobe specific flow volumes were prescribed at the outlets based on lung parenchyma lobe distribution reported in previous literature: 21% for the left upper lobe, 28% for the left lower lobe, 14% for the right upper lobe, 7% for the right middle lobe, and 30% for the right lower lobe [32]. It should be noted that airflow distribution among lung lobes can vary between individuals depending on anatomical features and breathing conditions; therefore, these values are used here as representative distributions derived from published study. This second boundary condition was used for sensitivity testing to verify that the conclusions were not dependent on outlet boundary assumptions.

**Table 1:** Whole Lung Deposition in all Five Models

Generations	Deposition in the lung (%)	Relative difference (%)	Standard deviation
(a) PIFR at 60 L/min, 3 µm particle size			
3	93.3	0.2	
4	94.2	1.2	
5	92.5	0.6	0.6
6	92.8	0.3	
7	93.1	/	
Mean deposition	93.1	/	
(b) PIFR at 60 L/min, 7 µm particle size			
3	8.5	65.6	
4	22.3	9.7	
5	17.5	29.1	6.4
6	21.6	12.6	
7	20.7	/	
Mean deposition	18.1	/	
(c) PIFR at 120 L/min, 3 µm particle size			
3	38.0	28.7	
4	30.2	43.3	
5	49.6	6.9	10.0
6	51.3	3.8	
7	53.3	/	
Mean deposition	44.5	/

**Table 2:** Airflow Partition Among Lung Lobes for the Five Airway Models (60 L/min)

Model	Inlet velocity (m/s)	Velocity at LU (m/s)	Percentage to inlet (%)	Velocity at LL (m/s)	Percentage to inlet (%)	Velocity at RU (m/s)	Percentage to inlet (%)	Velocity at RM (m/s)	Percentage to inlet (%)	Velocity at RL (m/s)	Percentage to inlet (%)
3	10.55	2.40	23	2.79	26	1.06	10	0.98	9	3.36	32
4	10.55	2.19	21	2.75	26	1.42	14	0.71	7	3.43	33
5	10.55	2.41	23	2.51	24	1.57	15	1.11	10	2.98	28
6	10.55	2.08	20	2.53	24	1.46	14	1.20	11	3.24	31
7	10.55	2.08	20	2.58	24	1.39	13	1.17	11	3.22	30

A turbulence intensity of 10% was prescribed at the inlet to represent moderately disturbed flow conditions typically observed in the upper respiratory tract during inhalation [33], which is also recommended in ANSYS guideline. The pressure–velocity coupling was managed using the SIMPLE algorithm with a convergence criterion of 10⁻^5^ [15, 22–25]. All simulations were conducted in ANSYS FLUENT, using double precision and parallel processing with six processors. All simulations began with a steady-state analysis, and the results were utilized as initialization conditions for subsequent transient simulations. The transient simulations covered an overall time duration of 4 s, with a time step size of 0.01 s.

### Mesh, Particle Number and Time Step Independence Tests

Mesh independence tests, injected particle number independence tests and time step independence tests were conducted using the lower airway model with seven bronchial generations. Mesh densities comprising 1.5 million, 3 million, 6 million, 12 million, and 24 million elements were tested. The results of the mesh independence tests were shown in Supplementary material (See Table SI1). 12 million mesh elements were used for all models, given that the maximum average velocity difference on L1 and L2 between the 12 million mesh elements and 24 million mesh elements was about 0.27% and the deposition difference in the lung was only 0.75%.

Four different injection files were utilised, each containing different particle numbers, and they are 2000, 4000, 8000, 16,000, and 32,000 particles. The particle deposition in the lung was then calculated for each of these cases, and the results are documented in Supplementary Information (See Table SI1). 16,000 particles were used for the simulations, given the small lung deposition difference of 0.18% between the 16,000 and 32,000 particle number cases.

The time step independence test was performed with five time-steps of 0.005 s, 0.01 s, 0.02 s, 0.04 s and 0.08 s. Results of the time step size independence tests (See Supplementary Information; Table SI1) show that the maximum average velocity difference on L1 and L2 between the 0.01 s and 0.005 s time step sizes were only 0.18% and the deposition difference in the lung was only 1.3%. Thus, A time step of 0.01 s was hence used for all the simulations.

### Governing Equations

The Reynolds number associated with each computational model was analysed using Eq. (1). The density of air (ρ) and its dynamic viscosity (μ) were assigned as 1.225 kg/m^3^ and 1.825 × 10^–5^ kg/ms, respectively. The velocity (v) was the peak flow rate for the transient flow cases, and characteristic length (L) was extracted from the inhaler diameter.

1 $$Re=\frac{\rho vL}{\mu }$$

The airflow within the human airway model is considered both incompressible and isothermal. The continuity and momentum equations are presented in Eqs. (2) and (3), respectively.

2 $$\frac{\partial {U}_{i}}{\partial {x}_{i}}=0$$

3 $$\frac{\partial {U}_{i}}{\partial t}+{U}_{j}\frac{\partial {U}_{i}}{\partial {x}_{j}}=-\frac{1}{\rho }\frac{\partial p}{\partial {x}_{i}}+\frac{\partial }{\partial {x}_{j}}[(\nu +{\nu }_{T})(\frac{\partial {U}_{i}}{\partial {x}_{j}}+\frac{\partial {U}_{j}}{\partial {x}_{i}})]$$

where U is the mean flow velocity (m/s), t is the time (s), p is the pressure (Pa), ρ is the density (kg/m^3^), and ν is the velocity vector (m/s).

As mentioned, the SST K-ω turbulence model [34, 35] was chosen as the numerical scheme to simulate internal flow, which is also consistent with many published work in similar field has used. The *SST k-ω* turbulence model combines the advantages of the k-ω and k-ε models through a blending function. This model captures near-wall turbulence effects using the k-ω formulation while transitioning to the *k-ε* formulation in the free stream, to improve accuracy in predicting flow separation and recirculation. The governing equations for turbulent kinetic energy (*k*) (see Eq. (4)) and specific dissipation rate (ω) (see Eq. (5)) are expressed as follows:

4 $$\frac{\partial (\rho k)}{\partial t}+\frac{\partial (\rho {u}_{i}k)}{\partial {x}_{i}}=\frac{\partial }{\partial {x}_{j}}\left[\left(\mu +{\sigma }_{k}{\mu }_{t}\right)\frac{\partial k}{{\partial x}_{j}}\right]+{P}_{k}-{\beta }^{*}\rho \omega k$$

5 $$\frac{\partial (\rho \omega )}{\partial t}+\frac{\partial (\rho {u}_{i}\omega )}{\partial {x}_{i}}=\frac{\partial }{\partial {x}_{j}}[\left(\mu +{\sigma }_{\omega }{\mu }_{t}\right)\frac{\partial \omega }{{\partial x}_{j}}]+\alpha \rho {S}^{2}-\beta \rho {\omega }^{2}$$

here *ρ* is the fluid density, *u*_*i*_ is the velocity component in the i-direction, *μ* and *μ*_*t*_ are the molecular and turbulent viscosities, respectively, *σ*_*k*_​ and *σ*_*ω*_​ are model constants, *P*_*k*_ is the production term for turbulent kinetic energy, *S* is the mean strain rate magnitude and *β*^*∗*^, *α* and *β* are empirical model coefficients. Work to validate the accuracy of this numerical scheme has been carried out in a previous study using particle image velocimetry (PIV) experiments, albeit with a lower airway model limited to three generations. Interested readers are encouraged to refer to the article for further details [36], which demonstrates that the numerical scheme is broadly capable of capturing the velocity field at the exit of the lower airway branch.

Particle transport and deposition in the airway models was simulated using a DPM model and a user-defined file where dispersion of the 16,000 particles was administered at the inhaler inlet. The equation that governs the particle transport simulation was derived from Eq. (6).

6 $${\rho }_{p}dp\frac{{d}^{2}xp}{d{t}^{2}}=\frac{3}{4}\rho {C}_{D}\left(v-vp\right)\left|v-vp\right|+{\rho }_{p}dpg$$

here *g* is the gravity vector (9.81 N/kg), p is the particle phase, ${\rho }_{P}$ is the particle density, *x*_*P*_ is the particle displacement, *d*_*P*_ is the particle diameter, *C*_*D*_ is the drag force coefficient, and *v* is the velocity vector (m/s).

## Results

### Effects of the Number of Airway Generations on Lung Particle Deposition

Whole-lung deposition was analysed across all five models. In this context, lung deposition includes particles deposited in the trachea and tracheobronchial tree, as well as escaped particles, which are assumed to represent total deposition in the deeper lung regions. The relative difference values are calculated by taking the absolute difference between the current generational complexity and that of the 7th-generation model, then dividing by the corresponding values in the 7th-generation model. Table 1 presents the total lung deposition for all five airway models with various generational complexity and aerosol delivery conditions (15 case studies in total).

The above results show that lung deposition efficiencies did not follow a systematic trend as the number of lower airway branches increased. For example, in simulations conducted at PIFR of 60 L/min, lung deposition was slightly higher in the 4th-generation lower airway model than in the third-generation lower airway model, but decreased again in the 5th-generation lower airway model. The difference is relatively small and is not observed consistently across the other cases (120 L/min condition). The variation may arise from local geometric differences when the airway model is truncated at the fourth generation, which can slightly modify airflow patterns and particle trajectories entering downstream branches. However, as the number of lower airway generations increased, the differences in lung deposition between models became progressively smaller, indicating that deposition values were approaching convergence.

The effect of particle size on lung deposition in models with different numbers of lower airway generations is demonstrated in Table 1a and 1b. As expected, cases simulated with smaller particles (3 µm) exhibited higher overall lung deposition compared to the corresponding cases simulated with 7 µm particles using similar lower airway geometries. The average deposition across all models that simulated 3 µm particles was 93.1%, with a small standard deviation of 0.6%. This suggests that simplified lung geometries may be sufficient for simulating particles of this size at a PIFR of 60 L/min. In contrast, Table 1b shows that the average lung deposition for 7 µm particles was significantly lower at 18.1%, with a standard deviation of 6.4% across models with different number of lower airway generations. While the reduced lung deposition efficiency for larger particles is well documented, these results suggest that increasing lung generational complexity is likely essential to accurately simulating the deposition of larger particles.

Table 1c shows the impact of flow rate on particle deposition efficiency in relation to lung geometry complexity. The results indicate that the average deposition across the airway models with different numbers of lower airway generations, simulated using 3 µm particles at the higher flow rate, decreases to 44.5%, with a standard deviation of 10%. These results suggest greater variability in lung deposition efficiency and highlight the influence of lung generational complexity when higher flow conditions are used. In addition, the differences in deposition between successive airway generations diminish exponentially. For example, the deposition difference is 3.8% between models with six and seven lower airway generations, whereas it is significantly higher for models with three and four generations—28.7% and 43.3%, respectively.

### Comparison of Particle Deposition in Specific Lower Airway Regions

This section analyses the effects of the number of lower airway generations on local particle deposition behaviour across three specific regions: Region 1 (Fig. 4a), Region 2 (Fig. 4b), and Region 3 (Fig. 4c). Particle deposition in these regions, for each model, was compared with that of the seven-generation lower airway model, simulated under the same flow conditions and particle sizes. The differences were analysed and expressed as percentages to facilitate data comparison. The first region is at the caudal end of the trachea, before airway bifurcation takes place (Fig. 4a). The second region is an airway branch located at the third generation (Fig. 4b). The third region is located at the distal terminus of the right lower bronchus (Fig. 4c). The rationale for selecting these airway regions is to delineate how deposition may be affected from the upper airway through to the deeper parts of the lung.

Figure 4a shows the percentage difference in particle deposition relative to the 7th-generation lower airway model at the tracheobronchial junction (carina), located at the caudal end of the trachea. The differences in deposition between the truncated models and the seven-generation airway reference model were most pronounced for the 3rd- and 4th-generation lower airway models, with differences exceeding 100%. As the number of lower airway generations increased, these discrepancies decreased rapidly and approached convergence by the model with six lower airway generations. This trend was consistent across all three groups of cases performed using different flow condition or particles sizes.

In Fig. 4b, which focuses on an airway branch located at the 3rd generation, the relative deposition differences for cases simulated at PIFR of 60 L/min with 3 µm particles remained notably large even in a fairly complex model with six lower airway generations. However, the deposition differences reduced dramatically when larger particles (7 µm) were simulated, falling within 10% of the reference case. This finding indicates that larger particles are less sensitive to airway model complexity at this region. The pronounced variability seen with 3 µm particles highlights their sensitivity to anatomical detail, as these smaller particles are more likely to reach and deposit in the distal lung regions. Furthermore, at higher flow rates, deposition differences for 3 µm particles diminished beyond the fifth generation, likely due to enhanced inertial impaction causing more particles to deposit in proximal airways, reducing their transport to distal regions.

In Fig. 4c, which anatomically shifts further downstream with each added generation, deposition differences were the most exaggerated. Models truncated at generation three or four overestimated deposition by as much as 800—1600% relative to the seven-generation case. These discrepancies were more extreme for the PIFR of 120 L/min case, indicating that under high-flow, high-inertia conditions, artificial termination of the airway leads to substantial overprediction of deposition due to premature particle impaction and reduced opportunity for distal transport and sedimentation. This strongly supports the notion that accurate modelling of distal generations is essential when evaluating deposition in peripheral regions, especially under realistic breathing or device-driven inhalation rates.

### Velocity Profiles

#### Comparison of Velocity Profiles Under Transient Flow Conditions

The velocity profiles across the cross-section of two airway regions (Fig. 3) are presented in Fig. 5. Figures 5a and b show the velocity profiles under transient flow conditions for peak inspiratory flow rates (PIFR) of 60 L/min and 120 L/min, respectively, captured at the peak flow time point (*t* = 0.5 s). The velocity profiles are generally pseudo-symmetrical for all cases. When examining differences between low and high peak flow rate cases at L2, the velocity profiles are largely similar across all models, aside from variations in velocity magnitude. Some discrepancies in the shape of the velocity profile are noted for the simplest models with three lower airway generations. The velocity profile at L1, located upstream of the lower airways and slightly caudal to the human pharynx, shows substantial variability across models with different lower airway geometry complexities. Notably, the velocity profiles for the 6th—and 7th—generation lower airway models remain closely similar.

#### Comparison of Velocity Profiles Under Steady Flow Conditions

Figure 6 compares the velocity profiles at L1 and L2 under constant inhalation simulations. For constant case, a steady inlet flow equal to the peak inspiratory flow rate of the transient simulation was applied. Result shows that the velocity profile at L2, just before the bifurcation, is not symmetrical, which is unlike those observed under transient flow conditions. In addition, although the velocity profile at L2 is fairly consistent across all models at PIFR of 60 L/min, the velocity profile at L1, upstream of the lower airway, again shows notable differences between cases, similar to the observation seen in the transient flow conditions in Fig. 6. The results also show that the velocity profiles are similar between the models with six and seven lower airway generations. With higher PIFR of 120 L/min, as shown in Fig. 7b, the velocity profile becomes more variable at L2, although the velocity profiles between the models with six and seven lower airway generations remain similar.

Results presented in Figs. 5 and 6 collectively suggest that the complexity of the lower airway can significantly affect upstream flow dynamics.

#### Airflow Partition Among Lung Lobes

In addition to velocity profiles, the airflow partition among the five lung lobes was analysed under the zero-outlet-pressure boundary condition for the 60 L/min inhalation case. The predicted flow distributions for the five airway models are summarised in Table 2.

The results show that the airflow partition remains relatively stable across the five airway geometries. For the most complete model (7th-generation lower airway model), the airflow distribution was 20% for the left upper lobe (LU), 24% for the left lower lobe (LL), 13% for the right upper lobe (RU), 11% for the right middle lobe (RM), and 30% for the right lower lobe (RL). These values are generally comparable with the lobe-specific distribution reported in published study [32] (21%, 28%, 14%, 7%, and 30%, respectively).

Minor differences between the predicted results and the reference values may arise from variations in airway geometry and truncation of distal bronchial generations in the present models, which can slightly alter downstream airway resistance and redistribute airflow among the lobar outlets. Nevertheless, the overall flow partition remains consistent across the five models, indicating that truncation of the airway geometry has a limited influence on lobar airflow distribution.

### Velocity Contours

Figure 7 further illustrates the impact of lower airway geometry on upstream flow dynamics. Velocity contours extracted at the peak inspiratory flow rate (PIFR) at two sections (P1 and P2) along the pharynx, along with L1, were analysed. Figure 7a shows the velocity contours, while Figs. 7b and c present the computed differences in velocity contours relative to the 7th -generation lower airway model, for PIFRs of 60 L/min and 120 L/min, respectively. The results suggest that the number of lower airway generations modelled may influence flow dynamics even at the level of the oral cavity and pharynx, particularly in high flow rate cases.

### Comparison Between Two Different Boundary Conditions Setup

**Fig. 8:**
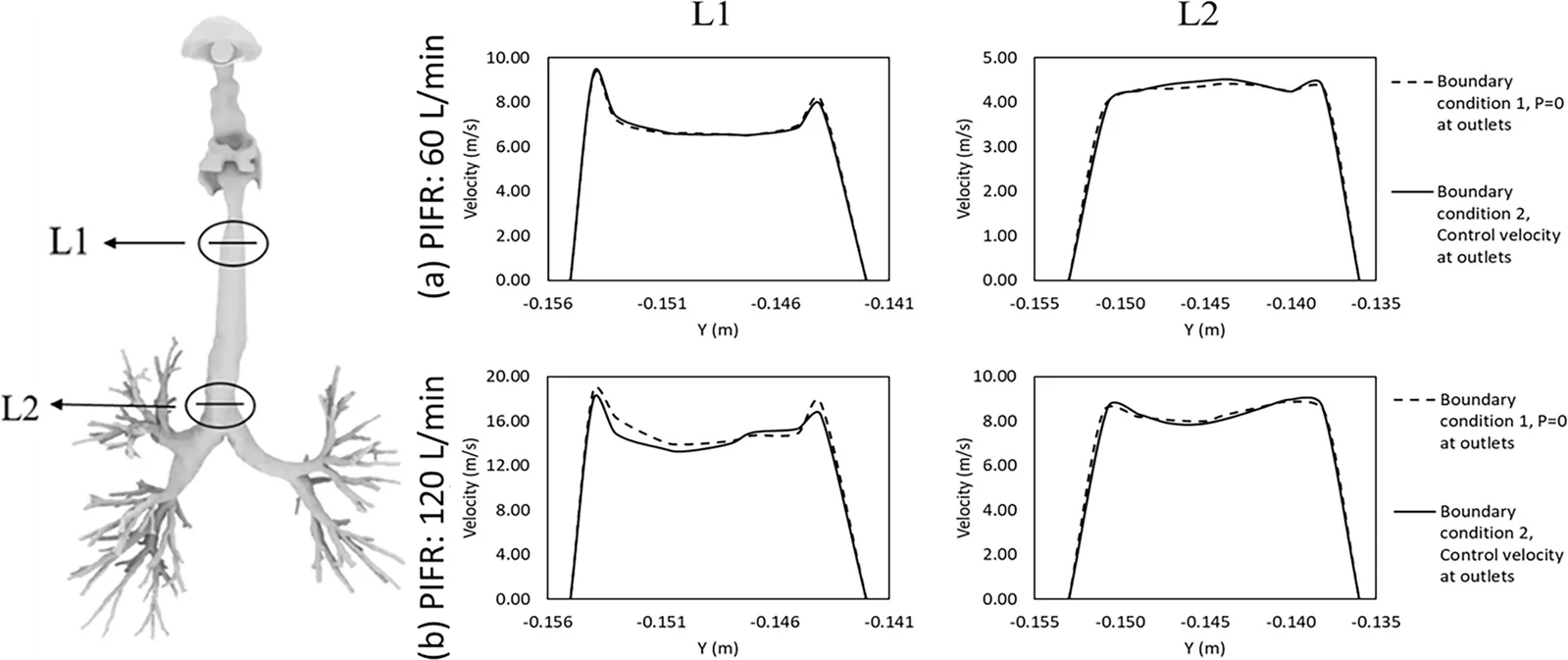
Comparison of velocity flow profile at L1 and L2 (see Fig. 3) between two boundary conditions under different transient flow rate conditions.

A plausible contention to the above observations is that flow boundary conditions may have been prescribed differently from those used in some existing models in the literature. In the current work, atmospheric pressure was prescribed at the airway outlets, as one objective was to identify the minimal geometrical detail that a physical lung replica should assume if it is to be enclosed within a chamber for *in-vitro* experiments. In the literature, some studies have instead prescribed outlet flow rates, such as in the majority of the work undertaken by Shang *et al*. [19], which are considered more physiologically relevant. For this reason, a comparative analysis of the velocity profiles using two different sets of boundary conditions was included as part of the study design. The results show that the choice of boundary conditions produced minimal differences in the velocity profiles at both L1 and L2, as well as in whole-lung particle deposition (Table 3 and Fig. 8).

**Table 3:** Comparison of the Lung Deposition in the Seventh-Generation Lower Airway Model Subjected to Different Boundary Conditions

Flow and particle size condition	Boundary condition	Deposition in the lung (%)	Differences (%)
PIFR 60 L/min, particle size: 3 µm	Pressure outlet, P = 0	93.1	0.4
	Velocity outlets	92.7	
PIFR 60 L/min, particle size: 7 µm	Pressure outlet, P = 0	20.7	3.1
	Velocity outlets	17.6	
PIFR 120 L/min, particle size: 3 µm	Pressure outlet, P = 0	53.3	2.5
	Velocity outlets	56.1

## Discussion

This study examined the impact of the number of lower airway generations on particle transport and deposition in the human respiratory airway. To the best of the authors' knowledge, this is the first study to discuss the potential discrepancies that simplified lower airway models may introduced. This work aims to provide experimental guidance on the appropriate physical lower airway replicas that could be manufactured to investigate drug delivery and deposition efficiency accurately and serves to offer insights that enhance the interpretation of existing and future research utilising simplified lower airway models.

An example based on the results obtained from this study suggest that lung deposition efficiency for 3 µm particles, simulated at a peak inhalation flow rate of 120 L/min, may have been underestimated by more than 10% when using a simplified model with only three lower airway generations. These discrepancies appear to be increasingly exacerbated when larger particles are simulated, where lung deposition efficiency could be underestimated by up to 65.6% compared to the reference, seven-generation lower airway model. Based on the findings, an airway model with at least six generations is likely necessary to more closely replicate the flow dynamics and deposition patterns observed in higher-fidelity airway models, at least under the flow rate and particle size conditions used in this study. The results also suggest that particle deposition studies that encompass only the human pharynx or models that include only the trachea with minimal lower airway generations may need to be interpreted in light of the findings presented in this current work.

The differences in flow dynamics and particle deposition observed between the models with different numbers of lower airway generations, as presented in this current study, may be attributed to differences in total outlet surface area and the systemic resistance introduced by additional airway generations. At least one study has suggested that the trachea [23], due to its long and rigid structure composed of cartilage rings, serves as a conduit that facilitates flow development. Notably, results from this current work reveal that the velocity profile is generally more consistent before the bifurcation than in the upstream region, demonstrating the function of the trachea, while also elucidating the complex flow dynamics being introduced upstream by simplified lower airway models. These findings suggest that a pseudo-complex respiratory tract model, incorporating at least six lower airway generations, may be necessary to accurately simulate and assess the efficacy of inhaled aerosol products. The importance of modelling the lower airway appears to have been underemphasised in the literature, where flow rate at the outlet is typically controlled to ensure relevant and appropriate flow dynamics, even when only sections of the airway are modelled in isolation.

The numerical scheme for the computational model used in this study, along with the parameters selected, has been widely adopted in published work within this field of research [15, 22–25]. As discussed in Methods, the model was validated in a previous study [36] using particle image velocimetry. Additional evidence supporting the accuracy of the results presented in this current study, which meaningfully aligns with the complexity of the models used, are as follows. Inthavong *et al*. [24] investigated air-particle dynamics in the tracheobronchial airway using spherical aerosol particles (3–10 μm) and a mean respiratory flow rate of 66 L/min. The axial velocity at S1 (prior to airway bifurcation), which corresponds to the L2 location in this study, exhibited a skewed parabolic shape identical to the velocity profile presented at L2 in the current work. Zhang and Finlay [23] studied the influence of tracheal features on particle deposition in the upper airway under steady-state flow conditions of 60 L/min. Their study utilised pseudo-monodisperse aerosol particles with mass median diameters ranging from 2.9 to 6.3 μm, and reported a lung deposition efficiency of 8%, which aligns closely with the 6.8% lower airway deposition observed in this current study. The slight disparity in results may be attributed to their use of a simplified, idealised lower airway model that comprises three lower airway generations. Given the high-fidelity airway models presented in this study, the deposition efficiency results should ideally correspond with existing *in vivo* studies to demonstrate the models’ accuracy. Yang *et al*. [37] performed SPECT (single-photon emission computed tomography) imaging on 21 subjects to assess *in vivo* particle deposition in the lower airway. Their results showed an average lung deposition of approximately 20 ± 12% for a PIFR of 50–70 L/min, which corresponds well with the case simulated in this study using 7 μm particles at a peak flow rate of 60 L/min, and in the model with seven lower airway generations.

Similar modelling challenges also persist in simulations of arterial blood flow and pressure, which require outflow boundary conditions that account for the influence of downstream vascular domains. To address this, researchers have developed coupled multidomain approaches that link analytical models of downstream domains with three-dimensional numerical models of the upstream vasculature [38]. In this framework, lumped parameter (0D) models are formulated as sets of ordinary differential equations that describe the temporal dynamics of variables within each compartment. Several studies have successfully coupled fully three-dimensional models of pulsatile blood flow with such reduced-order representations, including resistance-based boundary conditions. However, the parameters of these 0D models must be carefully calibrated prior to use. In contrast, comparable efforts in the respiratory system remain limited [39]. The present results provide insight into the potential need for parameter adjustment and highlight the importance of adopting alternative modelling strategies to account for downstream flow impedance effects.

There are several inherent limitations to consider when interpreting the results of this study. First, this study focuses on monodisperse particles, whereas aerosols in real-world scenarios are typically polydisperse. However, this choice was intentional and aimed to isolate the effect of particle size distribution, which could otherwise confound any observed differences in deposition attributable to the number of lower airway generations modelled. Another limitation is that the simulations were conducted using a single mouthpiece design and did not account for the fact that mouthpiece size can affect oropharyngeal geometry [40]. While a broader range of pharyngeal geometries and inhaler properties, such as resistance, could have been explored, these factors were intentionally held constant. Introducing such variability may dilute the clarity of the study’s conclusions, especially given the difficulty, to systematically interpret, and address variations associated with pharyngeal anatomical differences. While the use of different airway model geometries may alter specific results, such as absolute deposition efficiencies, the overall conclusions of this work are unlikely to be affected. As this work currently presents, it demonstrates the plausible effects based on the pharyngeal geometry used, which is anatomically and physiologically accurate. A further limitation is that only three test conditions were evaluated. However, it is worth noting that these conditions closely align with those commonly used in published studies in this field and were selected to facilitate comparison and interpretation. Despite the limited range of conditions, the study was able to adequately demonstrate clear and generalisable trends in lung deposition, velocity profiles, and flow contours as a function of lower airway complexity. Finally, the study did not report regional metrics such as the conducting-to-pulmonary (C/P) deposition ratio. While such metrics may offer additional detail, they were beyond the scope of this study, given that the primary aim of this current work is to assess and address the suitability of simplified airway geometries in capturing major trends in aerosol transport and deposition.

## Conclusion

This study investigated how lower airway geometry may impact airflow dynamics and particle deposition in the human respiratory tract. Results showed that simplified models, particularly those with fewer than six generations, may underestimate deposition efficiency and misrepresent upstream flow dynamics. Models with six or more generations showed convergence in outcomes, indicating that this level of complexity may be needed for accurate simulations. Collectively, these findings alluded to the importance of incorporating detailed lower airway models into both computational and experimental models to ensure realistic assessments of aerosol transport and drug delivery efficiency.

## Supplementary Information

Below is the link to the electronic supplementary material.

ESM 1 (DOCX 15.6 KB)

## Data Availability

The data that support the findings of this study are available from the corresponding author upon reasonable request.

## Abbreviations

CFD: Computational fluid dynamics
DE: Deposition efficiency
DPM: Discrete phase model
HRT: Human respiratory tract
PIFR: Peak inspiratory volume flow rate
PIV: Particle image velocimetry
SIMPLE: Semi-implicit method for pressure-linked equations
