# Supplementary material for: Effects of Airway Generation Depth on Particle Deposition Behaviour in the Main Respiratory Airways—A Computational Modelling Study
Source: Pharm Res. 2026 Apr 8;43(7):2407–21. doi: 10.1007/s11095-026-04084-6 (PMC13545025; doi:10.1007/s11095-026-04084-6)
Supplement: Supplementary file 1 [file 11095_2026_4084_MOESM1_ESM.docx]

# **Supplementary Information**

Table SI 1: Mesh size, injected particle number and time step independence test parameters.

| Mesh size (million) | Average velocity on L1 (m/s) | Average velocity on L2 (m/s) | Deposition in the lung (%) | Injected particle number | Deposition in the lung (%) | Time steps (s) | Average velocity on L1 (m/s) | Average velocity on L2 (m/s) | Deposition in the lung (%) |
| --- | --- | --- | --- | --- | --- | --- | --- | --- | --- |
| 1.5 | 6.0281 | 2.9063 | 40.9 | 2000 | 40.3 | 0.005 | 3.5263 | 7.1017 | 54.0 |
| 3 | 6.4133 | 3.201 | 47.1 | 4000 | 46.1 | 0.01 | 3.5198 | 7.0905 | 53.3 |
| 6 | 6.726 | 3.4899 | 51.6 | 8000 | 51.7 | 0.02 | 3.4989 | 6.7022 | 52.1 |
| 12 | 7.0905 | 3.5198 | 53.3 | 16000 | 53.3 | 0.04 | 3.207 | 6.3344 | 47.8 |
| 24 | 7.1102 | 3.5301 | 53.7 | 32000 | 53.4 | 0.08 | 2.9914 | 5.9771 | 45.1 |
